# Sacral Tarlov Cyst as a Rare Cause of Lower Limb Radiculopathy in a Child

**DOI:** 10.7759/cureus.90660

**Published:** 2025-08-21

**Authors:** Srujana V Venkata, David SK Mak, Jonis Michael L Esguerra, Sze Jet Aw, Lee Ping Ng, Sharon YY Low

**Affiliations:** 1 Department of Neurosurgery, National Neuroscience Institute, Singapore, SGP; 2 Neurosurgery, Cebu South Medical Center, Cebu, PHL; 3 Neurological Surgery, Vicente Sotto Memorial Medical Center, Cebu, PHL; 4 Department of Pathology and Laboratory Medicine, KK Women's and Children's Hospital, Singapore, SGP; 5 Neurosurgical Service, KK Women's and Children's Hospital, Singapore, SGP

**Keywords:** neuropathic pain, perineural cyst, radiculopathy, sacral tarlov cyst, spinal cyst

## Abstract

Tarlov cysts (TC) are uncommon perineural cysts that may arise anywhere along the spine, especially in the sacral region. To date, symptomatic TCs in children are very rare. Most of these benign lesions tend to be incidental discoveries during neuroimaging for other reasons. Nonetheless, they have been occasionally reported to cause sensorimotor-related neuropathy in the lower back, pelvis, legs, and urogenital system due to direct compression of the adjacent nerve roots. Management of adult TCs is well-described. In contrast, there is a paucity of contemporary literature on symptomatic TCs that are surgically treated in children. We report an unusual case of a paediatric patient who presents with lower limb radiculopathy secondary to large sacral TCs and discuss the management. A previously well 9-year-old female was referred for persistent lower back pain and left leg pain over a period of 2 months. There were no associated bladder or bowel symptoms. Clinical examination demonstrated a positive straight leg raise on the left and reduced sensation along the posterior left thigh, calf, foot dorsum, and toes. Magnetic resonance imaging (MRI) of her spine showed prominent, large perineural cysts surrounding bilateral S1 and the right S2 cauda equina nerve roots. Of note, there was associated bony expansion and scalloping of the sacral canal and corresponding sacral foramina. After a failed trial of conservative treatment, she underwent a laminotomy, wide fenestration, and excision of TCs. Postoperatively, her symptoms improved significantly. At 12 months’ follow-up, there was no recurrence of the patient’s lower back and leg symptoms. We describe an unusual case of symptomatic sacral TC in a child, whereby surgical treatment is a feasible treatment option in this age group.

## Introduction

Tarlov cysts (TC) or perineural cysts are rare and distinct clinicopathological entities in the lumbosacral region. They typically arise between the endoneurium and perineurium at the junction of the dorsal root ganglion and the posterior nerve root [1,2]. These spinal cysts tend to contain nerve root fibres and ganglion cells within their walls and cavities [3]. The presence of such anatomical nuances often makes complete surgical excision without morbidity challenging, incurring a higher rate of cyst recurrence and cerebrospinal fluid (CSF) leak. The prevalence of incidental TCs has been estimated to be up to 5% in adults versus 0.53% in the paediatric population [4,5]. To date, the aetiology of TCs remains unclear [6]. Several theories have been proposed, including congenital arachnoid proliferation along the nerve root sleeve versus secondary causes such as haemorrhage, trauma, infection, and inflammation [7,8]. Another accepted hypothesis is Paulsen’s ball-valve theory, whereby cysts arise due to one-way micro-communication with the subarachnoid space at the dural sleeve of the nerve root and subsequently enlarge via CSF hydrostatic and pulsatile inflow [4,9]. Occasionally, TCs may cause neurological symptoms either by compressing and stretching adjacent nerve roots or via direct pressure on adjacent periosteum or joint capsule [10-12]. In children, urinary incontinence has been temporally reported in the literature to be the most common presentation of symptomatic TCs. However, a timely, accurate diagnosis is challenging due to the wide spectrum of causative factors for paediatric urinary incontinence. While the various approaches for adult symptomatic TCs are well-described, most of the paediatric cases (albeit limited) are successfully managed with surgery. Overall, there is a paucity of publications relating to symptomatic TCs and their management in children. We present an unusual case of a paediatric TC presenting with lower back pain and lower limb radiculopathy to highlight surgical feasibility and outcomes in children. The natural history of TCs and aspects of their management are discussed in corroboration with contemporary literature.

## Case presentation

A previously well 9-year-old girl was referred to our clinic with a 2-month history of persistent aching lower back pain with sharp, shooting pain radiating down her left leg. At baseline, the patient rated a Numeric Rating Scale (NRS) of 5 out of 10 for her pain [13]. She was particularly troubled with symptom exacerbation upon standing and walking, with NRS increasing to 8 out of 10, and was unable to participate in sports or physical activity. There were no associated motor limb weakness or urinary symptoms. Prior to the referral to us, she was initially trialled on repeated courses of oral acetaminophen and non-steroidal anti-inflammatory medication for analgesia, combined with a 6-week outpatient period of physiotherapy. However, there was no significant pain improvement, and her physical activities remained limited. Various management options were explored, including continued conservative management with analgesia and lifestyle changes, nerve root injections, percutaneous cyst aspiration with glue injection, and microsurgical excision. Given the higher rate of recurrence associated with percutaneous cyst aspiration, a decision was made for definitive microsurgical resection of perineural cysts.

**Figure 1:**
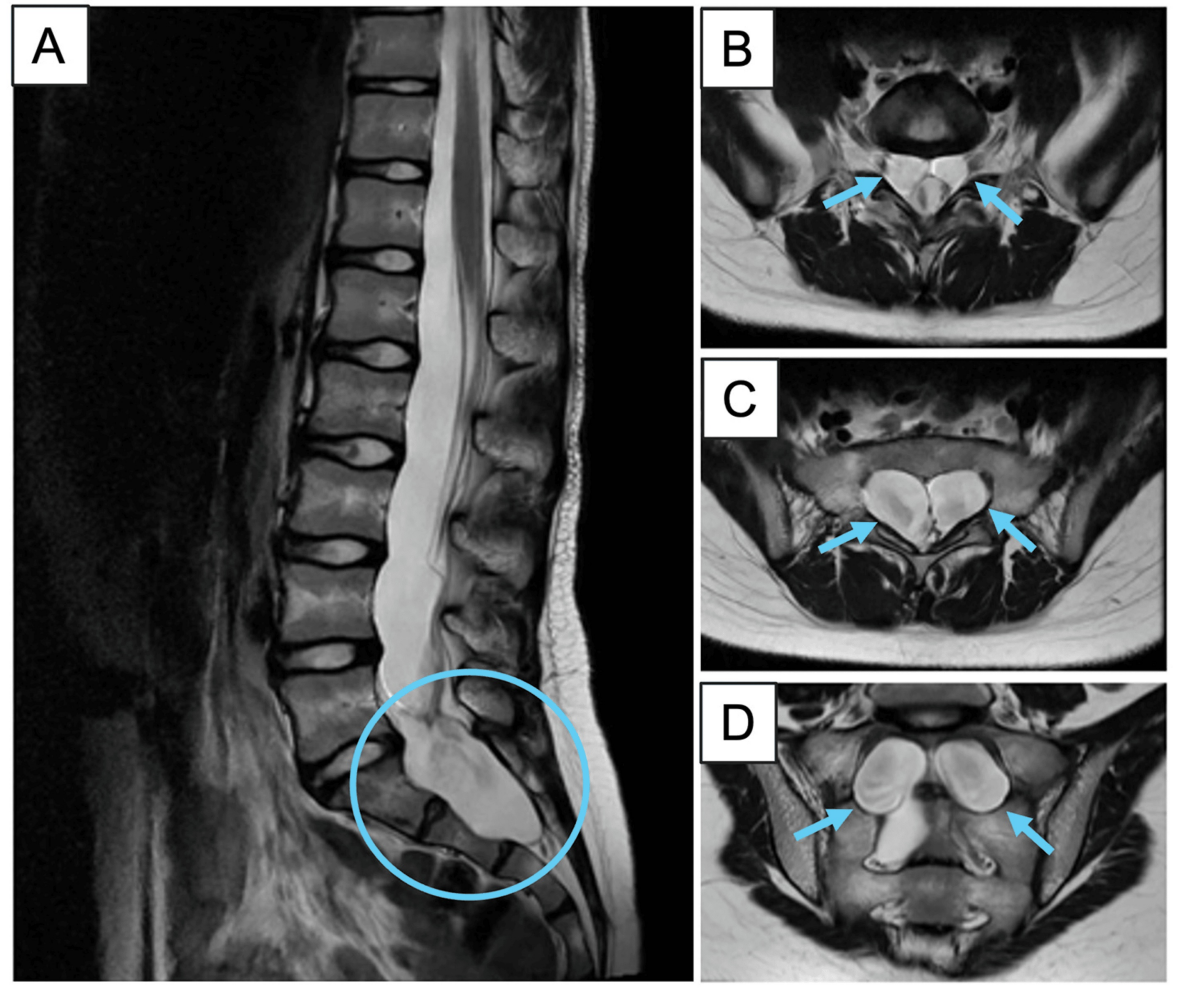
Representative T2-weighted MRI lumbosacral spine images in sagittal (A) and axial (B, C, D) directions. There are prominent perineural cysts (dimensions: right 5 × 5  × 2.3 cm and left 4 × 4 × 2 cm) surrounding bilateral S1 and right S2 cauda equina nerve roots (highlighted in light blue). MRI: magnetic resonance imaging

Clinical examination demonstrated a positive straight left leg raise, hypoesthesia involving the posterior left thigh, leg, and dorsum of the left foot and toes. Her gait was normal with full motor power along all myotomes of both lower limbs. In addition, lower limb reflexes were normal. She also had normal perineal and perianal sensation with normal anal wink. A bedside post-void residual urine (PVRU) examination did not demonstrate high residual urine volumes. Magnetic resonance imaging (MRI) of her lumbosacral spine reported large, bilateral perineural cysts surrounding the bilateral S1 and the right S2 cauda equina nerve roots. This was associated with expansion and scalloping of the sacral canal and corresponding sacral foramina. Owing to the significant size of both cysts, the adjacent neural foramina of interest could not be fully visualised. (Figure 1). Based on the patient’s clinical history, physical examination, and imaging results, the cause of her symptoms was likely neural compression from the large sacral cystic masses. At this juncture, the main differential diagnosis was that of perineural cysts (also referred to as TCs in this writing) arising from the S1 and S2 nerve roots, associated with adjacent bone scalloping. Other less probable considerations included lumbosacral arachnoid cysts, previously undetected meningoceles, and cystic schwannomas.

**Figure 2:**
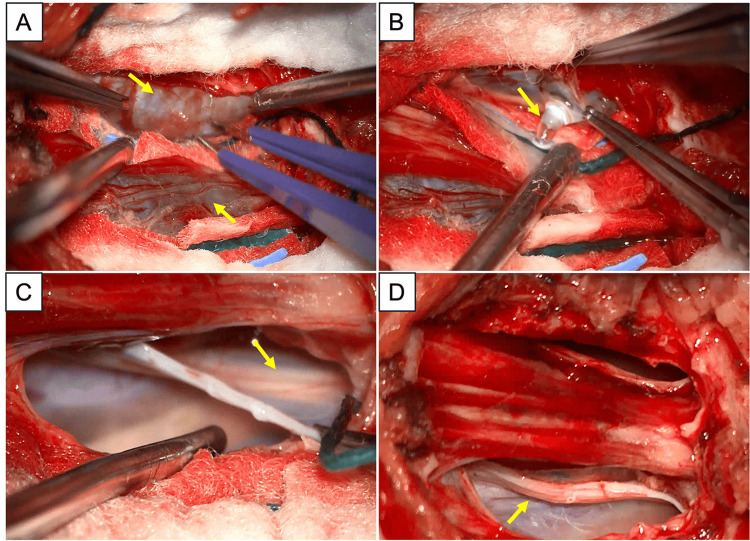
Representative intraoperative photographs that depict: (A) Bilateral intact sacral TCs after sacral laminectomy (yellow arrows); (B) Egression of cyst fluid from right TC after fenestration of TC wall (yellow arrow); (C) Visualisation of underlying right S1 nerve root after cyst fluid egression and part of TC wall removed (yellow arrow); and (D) Post-fenestration and TC wall excision shows a slightly swollen S1 nerve root (yellow arrow) and the sacral canal is adequately decompressed. TC: Tarlov cyst

A trial period of conservative treatment with analgesia and rest did not improve her symptoms. The decision was then made for neurosurgical intervention. The patient underwent an elective laminotomy, fenestration, and excision of bilateral S1/2 perineural cysts. She was placed in a prone position, and intraoperative neuromonitoring (IONM) with somatosensory evoked potentials (SSEP) and motor evoked potentials (MEP) was set up. Of interest, baseline intraoperative neuromonitoring showed a lower MEP threshold of left S2 to S4 levels in comparison to the corresponding right side, prior to skin incision. An incision was made, exposing from L5 to the midsacrum. Intraoperatively, the sacral bone was observed to be thinned out, more so on the left side than the right. A S1-2 laminotomy was carried out using a high-speed drill and with undercutting of the L5 laminae using Kerrison rongeurs. On removal of the dorsal wall of the sacrum up to the level of S2, the thecal sac was mobilised away to identify the perineural cysts attached to the length of the right S1 nerve root and left S2 nerve root. The identified TCs were microsurgically decompressed with a hook dissector and contents drained. Visually, the left S1 nerve root was noted to look slightly swollen at the end of the procedure-likely from prolonged compression. Monopolar stimulation and monitoring of muscle groups and the external anal sphincter were used to identify the pertinent S1-2 nerve roots along the cyst wall. Next, large sections of the TC walls were excised up to the point of visible perineurium and verified with monopolar stimulation. At the end of the surgery, the affected nerve roots were noted to be adequately decompressed, and there was no change from the baseline IONM readings. A collagen sealant graft was used to overlay the nerve root defect, followed by replacement of the sacral bone flap. To prevent CSF leak, a water-tight layered closure of the fascia and skin was performed (Figure 2).

**Figure 3:**
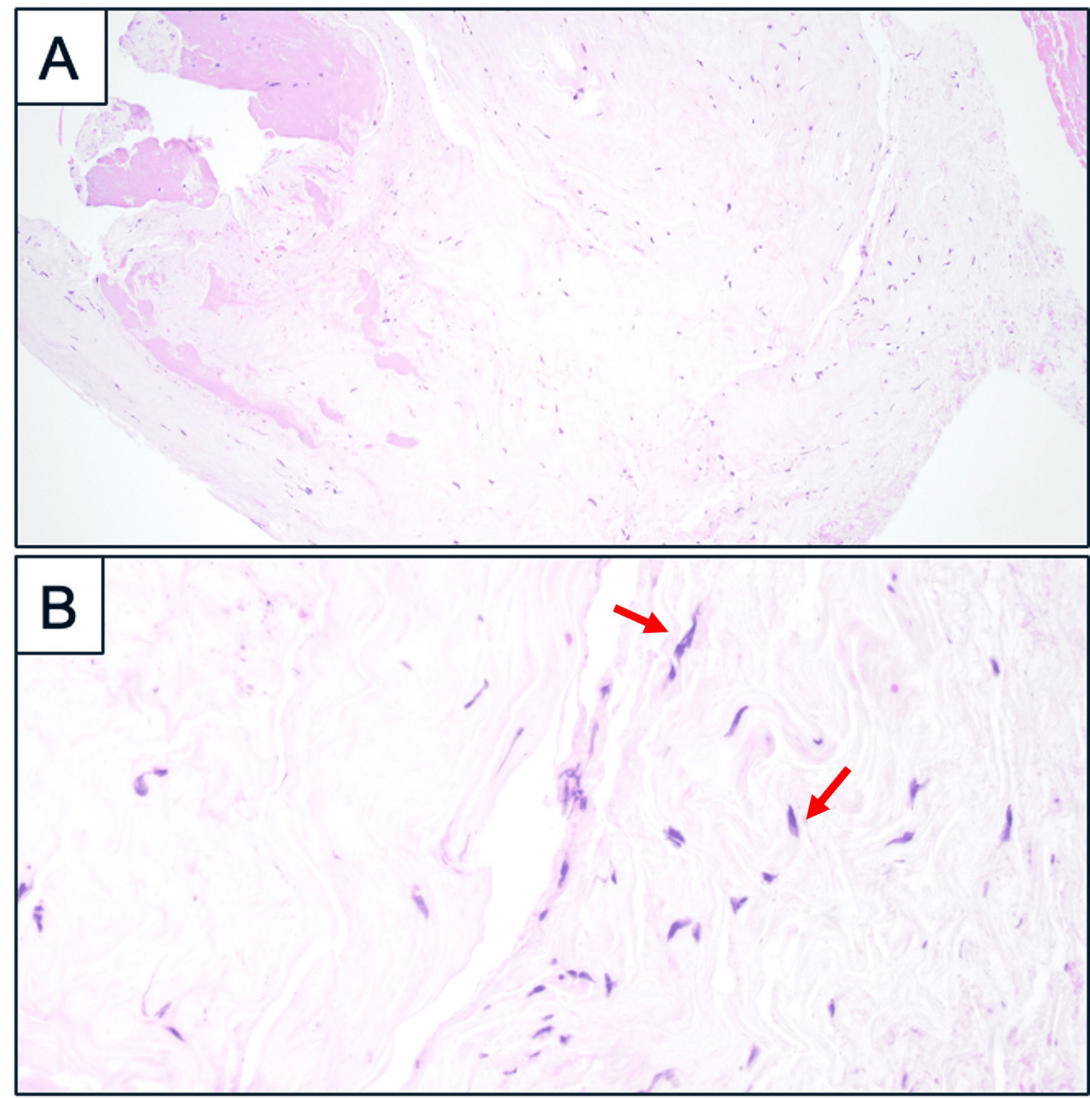
Haematoxylin and eosin-stained images of the excised TC walls at (A) Low power view shows hypocellular fragments of pale connective tissue with bland spindle cells (×10); and (B) High power view showing spindle cells featuring elongated, dark nuclei, compatible with perineural cells (×40). Examples of these perineural cells are highlighted with red arrows. TC: Tarlov cyst

Postoperatively, the patient was instructed to remain supine for 72 hours. A successful trial off the catheter was achieved with minimal postoperative residual urine volume. The child was discharged on the 6th postoperative day with minimal lower back pain symptoms, a pain score of 2/10, and resolution of radicular pain. Histopathology finding reveals the layers of flattened cells surrounded by a basement membrane and collagen fibres in keeping with fibroconnective tissue compatible with a perineural cyst wall. (Figure 3). At 3 months’ follow-up, the patient presented with a painless, soft swelling in her lower back region. The overlying wound was well-healed. She was clinically well with no new neurological or constitutional symptoms. Repeat MRI showed findings consistent with a pseudomeningocele. The MRI also demonstrated adequate decompression of the bilateral S1/2 nerve roots. A lumbar corset was provided, and subsequent outpatient reviews showed improvement of the lumbar swelling. At 12 months’ follow-up, the child’s initial presenting complaints of neuropathic pain symptoms gradually improved. To aid her functional recovery, a referral to a Paediatric Pain Specialist was made to provide holistic strategies to cope with her prolonged symptoms from a biopsychosocial perspective.

## Discussion

Sacral TCs tend to arise in the intersection of the dorsal root ganglion and posterior nerve root between the endoneurium and perineurium. Based on current understanding, TCs larger than 1 to 1.5 cm in diameter may cause clinical symptoms from local nerve root compression [11]. Currently, no specific criterion is available for differentiating between asymptomatic versus symptomatic TCs [14,15]. Diagnosis of TCs is solely based on imaging findings, and clinical correlation between the scans and each patient’s symptomology is required. The latter may include sensory disturbances, motor deficits, and dysfunction related to the autonomic system [16]. As previously mentioned in our case description, it is important to consider other differentials for the neuroimaging findings. Examples include other types of meningeal cysts or arachnoid cysts that do not involve the nerve fibres within the cyst walls, cystic schwannomas, whereby the cyst walls are typically contrast-enhancing, and finally, dural ectasia, a condition where there is widening and ballooning of the dural sac [17-21]. The latter can contribute to pain and neurological dysfunction but tends not to occur in the sacral region or involve the perineurium. Of interest, dural ectasia and TCs have been reported to be more prevalent in children with connective tissue disorders such as Marfan’s Syndrome, Ehlers-Danlos Syndrome, and Loeys-Dietz Syndrome [22,23].

Broadly speaking, treatment of symptomatic TCs can be divided into conservative, medical, and invasive therapies, which can be further subdivided into percutaneous or open surgical techniques [24-26]. For invasive procedures, the presence of viable nerve root fibres and ganglion cells within the cyst walls may result in injury to these neural structures during the process of intervention. Other significant risks associated with invasive treatments include genitourinary disturbances, CSF leak leading to secondary infection, and TC recurrence [6]. To date, several approaches have been described in adult literature with no consensus on the best treatment modality. Examples include CSF diversion to equalise CSF pressure between the cephalad thecal sac and perineural cyst with a cysto-arachnoid or lumbo-peritoneal shunt; percutaneous cyst aspiration and/or fibrin glue injection; and microsurgical fenestration or excision [20,25-28]. Overall, TC recurrence rates are estimated at less than 10% [8], with more than 70% obliteration estimated in adult literature with a combination of microsurgical excision and closure with fibrin glue [6]. Regardless of intervention, the rates of symptom improvement have been found to be widely variable, with components of psychological sequelae reported [8,23]. Adult patients with TCs may report depression, debilitating neuropathic pain, psychosocial issues, and even sexual disorders [29-31]. To reiterate, most TCs are incidental findings and only a minority are clinically concerning [24,25,32]. Hence, the decision for intervention relies on the managing clinician’s expertise to correlate the patient’s TC and presenting symptoms in the presence of other comorbidities, if relevant. Put together, judicious patient selection is critical as most TCs are incidental findings and may not be the only cause of an individual’s complaints.

At the time of this writing, literature regarding symptomatic TCs that are surgically treated remains sparse in the paediatric population [12,22,33-36]. Nevertheless, a recent meta-analysis reinstates the role of open surgery as a feasible option for sustained, long-term resolution of symptoms [37]. Although likely extrapolated from adult experience, a curated list of anecdotal paediatric cases demonstrates that surgically managed TCs demonstrate good clinical outcomes (Table 1) [6,12,22,33-36,38,39]. In this collective group, surgical success was reported across all studies. Recurrence occurred in 10%, which is also consistent with an 8.5% recurrence rate described in the adult literature [37]. The key factor for recurrence is recognised to be due to incomplete excision of the TC wall, precluded by involvement of neural elements. Next, CSF leak with the development of pseudomeningocele has been reported in 3 to 9% of adult series [23]. Here, primary closure is often difficult. To avoid this complication, the use of autologous muscle/fat grafts, dural grafts, fibrin sealant, and or replacing the overlying sacral bone has all been proposed [37]. Pertaining to our case, we encountered a CSF leak with a pseudomeningocele 3 months postoperatively, despite using a dural sealant, replacing the sacral bone, and postoperative bed rest to facilitate healing before mobilisation. We are fortunate that the pseudomeningocele improved with conservative measures. Nonetheless, this aspect of our case underscores the importance of a water-tight fascial closure to prevent wound breakdown and consequent CSF infection.

**Table 1:** Curated list of paediatric TCs that were surgically managed. Y: years old; m: months old; M: male; F: female

#	Author	Age/Gender	Level	Main Symptom	Treatment	Follow-up (Months)	Outcome
1	Elsawaf et al., 2016 [6]	7Y/M	S1/2	Nocturnal enuresis	Surgical excision	27	Improved
		7Y/F	S2/3	Nocturnal enuresis	Surgical excision	77	Improved
2	Dayyani and Zabihyan, 2019 [38]	8 m/F	L2	Abdominal distension, instability in the sitting position	Surgical Excision	6	Improved (had cystoperitoneal shunt for recurrence)
3	Mijalcic et al., 2019 [33]	7 Y/M	S2	Urinary Incontinence	Surgical excision	2	Improved
4	Yoshioka et al., 2021 [12]	7 Y/F	S3	Urinary and faecal incontinence	Rotation flap technique	24	Improved
5	Otto and Braun, 2024 [31]	5 Y/M	L4-S5	Nocturnal enuresis	Surgical excision	48	Improved
6	Shimauchi-Ohtaki et al., 2022 [35]	13 Y/F	S3	Severe constipation and urinary incontinence	Surgical excision	60	Improved
7	Shams et al., 2022 [22]	11 Y/M	S1/2	Double incontinence	Surgical Excision	18	Improved (underwent interval filum sectioning)
8	Huang et al., 2023 [39]	8 Y/F	S1-3	Chronic foot wound with hypoesthesia	Surgical excision	6	Improved
9	Siller et al., 2024 [34]	15Y/F	S1-3	Double incontinence	Surgical excision	6	Improved
10	Our case, 2025	9Y/F	S1/2	Left lower limb radiculopathy	Surgical excision	12	Improved

Separately, we note that urinary incontinence is the most common presenting symptom in children. Sacral TCs are frequently asymptomatic and regarded as incidental lesions in adults. For children wherein the spinal canal is anatomically narrower, a pre-existing cyst in the same restricted space may have a higher tendency to compress on the adjacent sacral nerve roots. Consequently, affected patients can present with urinary symptoms, especially in younger children [36,40]. Our case was unique in presentation with lower back pain and localised lower limb radiculopathy due to the cyst growing and extending superiorly and stretching the S1 nerve root. This is a clinical presentation that has not previously been described in the literature and in older children. In our case, surgical management was shown to be effective in specifically improving her symptoms. Percutaneous fibrin glue injection may be a less invasive alternative, but the caveat is its comparatively higher recurrence rates (i.e., 20% vs 8%) as reported in adult series [23]. In the context of our patient, we believe a future-proof approach for long-term symptom alleviation is especially relevant in a child with an extended lifespan. Furthermore, we are cognizant that persistent symptoms from the untreated paediatric TCs may impact school performance, participation in sporting activities and mental health wellness as highlighted in our case. Under such circumstances, a concerted approach from a multidisciplinary team to coordinate various aspects of care is paramount for optimal outcomes [23,41-43]. Future efforts to incorporate objective measurements for peri-operative physical functioning and disability, age-appropriate pain assessment scores and longitudinal quality of life feedback outcomes are also necessary.

## Conclusions

Symptomatic sacral TCs are very rare in the paediatric population. Herein, we report a unique presentation of large paediatric TCs with lower back pain and lower limb radiculopathy, in contrast to previous reports of mainly isolated urinary symptoms in children. Independent of age, these lesions can cause lumbosacral root compression leading to significant neuropathy and or urogenital symptoms that impact quality of life. Congruent with what is reported in adult literature, surgical intervention can be an effective option to decompress and resect these lesions with good results in this younger age group. Nonetheless, we are cognizant that extended follow-up is crucial to determine the long-term efficacy of this management approach. In the setting of limited established guidelines for management, we believe that an individualised approach after consensus with each patient, caregivers and a multidisciplinary team is the way forward for now. Overall, this case adds to the limited body of literature reports for a better understanding of TCs in children. We advocate collaborations with the international community to establish paediatric TC management guidelines and in-depth research on the aetiology of these challenging lesions.
